# Supplementary material for: Hybrid Models and Biological Model Reduction with PyDSTool
Source: PLoS Comput Biol. 2012 Aug 9;8(8):e1002628. doi: 10.1371/journal.pcbi.1002628 (PMC3415397; doi:10.1371/journal.pcbi.1002628)
Supplement: Text S4 — Complete source code for the PyDSTool package (version 0.88.120504). Includes API documentation and help files linking to web pages. This file is identical to the current public release on Sourceforge.net. (ZIP) [file pcbi.1002628.s004.zip › PyDSTool/html/PyDSTool.Toolbox.optimizers.step-pysrc.html]

xml version="1.0" encoding="ascii"?


PyDSTool.Toolbox.optimizers.step


| Home | Trees | Indices | Help | PyDSTool |
| --- | --- | --- | --- | --- |

Package PyDSTool ::
Package Toolbox ::
Package optimizers ::
Package step

[hide private]

[frames] | no frames]

# Source Code for Package PyDSTool.Toolbox.optimizers.step

```
 1   
 2  # Matthieu Brucher 
 3  # Last Change : 2007-08-31 13:42 
 4   
 5  """ 
 6  Module containing every step use to lower a cost function 
 7   
 8  Steps : 
 9    - GradientStep 
10      - compute a step based on the gradient of the function 
11    - CWConjugateGradient 
12      - Crowder-Wolfe conjugate gradient 
13    - DYConjugateGradient 
14      - Dai-Yuan conjugate gradient 
15    - DConjugateGradient 
16      - Dixon conjugate gradient 
17    - FRConjugateGradient 
18      - Fletcher-Reeves conjugate gradient 
19    - PRPConjugateGradient 
20      - Polak-Ribiere-Polyak conjugate gradient 
21    - FRPRPConjugateGradient 
22      - Fletcher-Reeves modified Polak-Ribiere-Polyak conjugate gradient 
23   
24    - NewtonStep 
25      - computes a step based on the hessian and the gradient of the function 
26    - MarquardtStep 
27      - computes a step based on the Marquardt modified hessian and the gradient of the function 
28    - GoldsteinPriceStep 
29      - computes a step based on the Goldstein-Price Newton modification 
30    - GoldfeldStep 
31      - computes a step based on the Goldfeld Newton modification 
32    - DFPNewtonStep 
33      - computes a step based on the Davidson-Fletcher-Powell approximation of the hessian 
34   
35    - PartialStep 
36      - decorator for another step but uses only part of this step 
37    - RestartPeriodicallyConjugateGradientStep 
38      - decorator for a conjugate gradient step that restarts the conjugation each n iterations 
39    - RestartNotOrthogonalConjugateGradientStep 
40      - decorator for a conjugate gradient step that restarts the conjugation if the last gradients are not orthogonal enough 
41   
42    - LocalBruteForce1Dstep 
43      - local brute force step in 1D (experimental) 
44  """ 
45   
46  from gradient_step import * 
47  from conjugate_gradient_step import * 
48   
49  from newton_step import * 
50  from marquardt_step import * 
51  from quasi_newton_step import * 
52  from goldfeld_step import * 
53  from goldstein_price_step import * 
54   
55  from partial_step import * 
56  from restart_conjugate_gradient import * 
57   
58  from local_brute_force_1dstep import * 
59   
60  step__all__ = ['GradientStep', 
61                 'CWConjugateGradientStep', 'DYConjugateGradientStep', 'DConjugateGradientStep', 'FRConjugateGradientStep', 'PRPConjugateGradientStep', 'FRPRPConjugateGradientStep', 
62                 'NewtonStep', 'MarquardtStep', 'GoldsteinPriceStep', 'GoldfeldStep', 
63                 'PartialStep', 
64                 'RestartPeriodicallyConjugateGradientStep', 'RestartNotOrthogonalConjugateGradientStep', 
65                 'LocalBruteForce1DStep'] 
66   
67  __all__ = step__all__ 
68
```

  


| Home | Trees | Indices | Help | PyDSTool |
| --- | --- | --- | --- | --- |

|  |  |
| --- | --- |
| Generated by Epydoc 3.0.1 on Fri May 4 15:24:19 2012 | http://epydoc.sourceforge.net |
